# Increased Prefrontal Cortical Thickness Is Associated with Enhanced Abilities to Regulate Emotions in PTSD-Free Women with Borderline Personality Disorder

**DOI:** 10.1371/journal.pone.0065584

**Published:** 2013-06-05

**Authors:** Hannah Bruehl, Sandra Preißler, Isabella Heuser, Hauke R. Heekeren, Stefan Roepke, Isabel Dziobek

**Affiliations:** 1 Department of Psychology of Emotion and Affective Neuroscience, Freie Universität Berlin, Berlin, Germany; 2 Department of Biological und Clinical Psychology, Friedrich Schiller University of Jena, Jena, Germany; 3 Department of Psychiatry, Charité – Universitätsmedizin Berlin, Berlin, Germany; 4 Cluster Languages of Emotion, Freie Universität Berlin, Berlin, Germany; University of Granada, Spain

## Abstract

Previous studies suggest that amygdala, insula and prefrontal cortex (PFC) disintegrity play a crucial role in the failure to adequately regulate emotions in Borderline Personality Disorder (BPD). However, prior results are confounded by the high rate of comorbidity with Posttraumatic Stress Disorder (PTSD), which itself has been associated with changes in frontolimbic circuitry. We thus scrutinized the link between PFC, amygdala, insula, and the ability to regulate emotions, contrasting 17 women with BPD without comorbid PTSD to 27 non-clinical control women and in addition to those with BPD and PTSD (n = 14). BPD women without PTSD, but not those with comorbid PTSD, had increased cortical thickness in the dorsolateral PFC (DLPFC) in comparison to control women. Furthermore, cortical thickness in the DLPFC of BPD women without PTSD positively correlated with emotion regulation scores and furthermore was positively associated with amygdala volume, as well as cortical thickness of the insula. Our findings highlight the importance of disentangling the impact of BPD and PTSD on the brain and suggest possible compensatory mechanisms for the impaired emotion regulation in BPD women without PTSD.

## Introduction

Borderline Personality Disorder (BPD) is a severe psychiatric disorder, characterized by abnormalities in interpersonal, behavioral and emotional functioning. It has been postulated that of these abnormalities, the inability to adequately regulate emotions constitutes the key feature of BPD [1]. The current view regarding the neural correlates of emotion regulation holds that it involves a network of regions encompassing the hippocampus, amygdala and prefrontal cortex (PFC) [2]–[7]. In accordance with both this suggested network and the view of BPD as a disorder of emotion regulation, most neuroimaging studies with BPD patients have yielded structural and functional abnormalities in the hippocampus, amygdala and PFC [8], and in addition, in the insula [9].

There is considerable heterogeneity in the findings, however, particularly with respect to alterations in brain structure. This might be due to the heterogeneity of the BPD patient groups themselves.

One confounding factor in the studies to date is the variable number of patients with Posttraumatic Stress Disorder (PTSD) included in virtually all of the studies on structural alterations in BPD. PTSD is comorbid with BPD in up to 50% of cases [10], [11] and there is considerable overlap with respect to brain structural alterations between the two disorders [8], making PTSD comorbidity a key issue for BPD research. For example, smaller hippocampal and, to some degree, smaller amygdalar volumes have been frequently found in BPD [12], [13], as well as in PTSD [14], although there are also accounts of no differences in amygdala volume [15], or even larger grey matter density in the amygdala [16] in BPD in comparison to non-clinical controls.

To date, only one study directly compared both BPD patients with and without PTSD to non-clinical controls on hippocampal and amygdalar volumes [17]. The authors found that only those patients with co-morbid PTSD had smaller hippocampal volumes than non-clinical controls. Moreover, a recent metaanalysis on hippocampal volumes in studies with BPD patients with PTSD and studies with patients without PTSD showed that only those with comorbid PTSD had clear bilateral volume reductions [18], [19], thereby highlighting the importance of disentangling the respective disorders’ effects on brain structure.

Apart from the hippocampus and amydgala, the PFC has been implicated in disturbed emotion regulation in BPD. Regarding structural alterations of the PFC, findings in BPD patients vary with the age group investigated. In adults with BPD, relative volumetric reductions and decreased gray matter density in the orbitofrontal and anterior cingulate cortex have been reported [16], [20], [21], although there is also an account of no prefrontal gray matter density changes detected anywhere in the PFC of adults [22]. Concerning the dorsolateral PFC (DLPFC) in BPD, one study found decreased gray matter density in teenagers [23], however, in adults, two studies using manual tracing did not detect alterations in the DLPFC [21], [24]. Paralleling the overlap of structural findings between BPD and PTSD with respect to the hippocampus and amygdala, volumetric reductions in orbitofrontal and DLPFC, as well as the ventromedial PFC have been described in adult patients with PTSD [25], [26].

Despite the role of hippocampus, amygdala and PFC for emotion regulation, previous structural neuroimaging studies in BPD did not link the observed alterations in these structures to emotion regulation abilities. Functional neuroimaging studies, in contrast, have demonstrated abnormalities in response to emotional stimulus material.

A recent metaanalysis of functional MRI studies with BPD patients suggests greater activations in the insula and posterior cingulate cortex but less activation in the amygdala, subgenual ACC and bilateral DLPFC when processing negative emotions [9]. However, other studies, that were not included in that metaanalysis, have reported greater amygdala activation in BPD patients as compared to controls in response to negative emotional stimulus material [27], [28]. Furthermore, the enhanced amygdala activation correlates with self-reported deficits in emotion regulation [28].

In addition to the aberrant amygdala and increased insula response, as mentioned above, diminished DLPFC recruitment when processing negative emotions [9] and prefrontal hypometabolism [29], [30] have been described in BPD patients.

Taken together, the neuroimaging findings have led to the hypothesis that a dysfunctional fronto-limbic network underlies emotional dysregulation in BPD [8], [13], [31], [32]. There is substantial overlap between the pattern of neural abnormalities in BPD and PTSD with respect to the amygdala, insula and PFC [33], [34]. Moreover, inverse rCBF coupling between the amygdala and the PFC has been reported in PTSD [35]. Therefore, the hypothesis has been put forward that PTSD, similar to BPD, is characterized by abnormal amygdala functioning and defective regulation from a hypoactive PFC [36]. Thus, given the high comorbidity and great overlap in neuroimaging findings between studies on BPD and PTSD, PTSD has to be considered a significant confound when identifying brain structural alterations in BPD.

Therefore, the primary aim of this study was to identify brain regions that are affected by BPD without the impact of comorbid PTSD. To this end, we compared patients with BPD with and without comorbid PTSD to non-clinical controls and among one another. Second, we wanted to scrutinize whether the brain regions thus identified would be related to emotional dysregulation in a group of patients with BPD. Based on prior findings, we hypothesized that we would find abnormalities in the PFC, amygdala and the insula. We chose cortical thickness as our major means of assessing prefrontal brain integrity, given that it might be more sensitive to subtle changes than voxel-based morphometry, which involves confounding factors introduced by normalization [37]. Additionally, cortical thickness measurements have been validated as being similarly sensitive as manual tracing [38], however they are also able to detect subtle changes that a priori regions of interest cannot. In addition to prefrontal brain integrity, we also assessed the *a priori* defined insula by means of cortical thickness measurements. For the amygdala we chose to follow an automated volumetric approach, which has recently been shown to be a reliable measure for subcortical limbic structures [38].

## Methods

### 2.1 Participants

Thirty-one unmedicated women with a diagnosis of BPD (average age: 28 years) and 27 non-clinical control women (NC, average age: 27 years) participated in the study. All BPD patients were inpatients admitted for specialized BPD treatment from a waiting list; all BPD patients had outpatient status before admission; none was transferred from another institution to our hospital or admitted for acute care. All NC women were recruited via advertising in local media outlets and were reimbursed for their participation upon completion of the study. The NC women were selected to be of similar age and to have a comparable fluid IQ (see below) as the Borderline women. Fourteen of the patients with BPD also had a diagnosis of current PTSD according to DSM-IV criteria. Borderline women with PTSD did not differ from Borderline women without PTSD with respect to the frequency of other comorbidities (see Table 1). Axis I and II diagnoses were made using the Mini–International Neuropsychiatric Interview, M.I.N.I. [39] and the Structured Clinical Interview for DSM-IV Axis II Disorders, SCID II [40]. All participants were free from psychotropic medication for at least two weeks before entering the study. A current neurological or medical disorder that could affect cerebral metabolism and an IQ below 80 served as exclusion criteria. Patients with BPD were not included in the study if they had current anorexia nervosa, psychotic, or substance use disorder within the past six months.

**Table 1:** Patients (BPD) and nonclinical controls (NC) group characteristics.

	NC (N = 27)	All BPD patients (N = 31)	BPD without PTSD (N = 17)	BPD with PTSD (N = 14)
Measure	MeanSD	MeanSD	MeanSD	MeanSD
Age (in years)	28.28.2	26.77.9	26.88.7	26.67.0
IQ (LPS subtest 4)	122.711.4	118.9012.1	119.8811.2	117.7913.4
IQ (WST)*	102.59.1	96.19.7	97.79.1	94.410.3
Emo. regul. (SEE) ** ^,^ #, ##	13.192.66	9.232.21	9.532.15	8.852.30
BSL mean score** # ##	0.720.58	2.320.75	2.300.88	2.340.58
*Axis I comorbidity*				
Major Depression (lifetime)	n.a.	38.7%	35.3%	42.9%
Major Depression (current)	n.a.	0%	0%	0%
Dysthymia	n.a.	32.3%	23.5%	42.9%
Bipolar I Disorder	n.a.	0%	0%	0%
Panic Disorder	n.a.	6.5%	5.9%	7.1%
Agoraphobia	n.a.	22.6%	29.4%	14.3%
Social Phobia	n.a.	16.1%	11.8%	21.4%
Obsessive Compulsive Disorder	n.a.	6.5%	0%	14.3%
Bulimia Nervosa	n.a.	19.4%	23.5%	14.3%
*Axis II comorbidity*				
Schizoid PD	n.a.	0%	0%	0%
Paranoid PD	n.a.	0%	0%	0%
Schizotypal PD	n.a.	0%	0%	0%
Histrionic PD	n.a.	0%	0%	0%
Narcissistic PD	n.a.	3.2%	5.9	0%
Antisocial PD	n.a.	6.5%	11.8	0%
Obsessive compulsive PD	n.a.	6.5%	11.8	0%
Avoidant PD	n.a.	29%	23.5	35.7%
Dependent PD	n.a.	3.2%	0%	7.1%

Abbreviations: LPS = Leistungspruefsystem (fluid intelligence), WST = Wortschatztest (crystallized intelligence), SEE: Subjective Experience of Emotions, BSL = Borderline Symptom List, PD = personality disorder.

** *NC vs. BPD p<0.001.*

* *NC vs. BPD p<0.05.*

# *NC vs. BPD without PTSD p<0.05.*

## *NC vs. BPD with PTSD p<0.05.*

100% of the BPD group with, and 100% of the BPD group without PTSD reported having experienced at least 1 or more traumatic event and they did not differ on overall traumatic experience (λ = 0.564, F = 0.985, df = 11, *p* = 0.501) based on the Posttraumatic Stress Diagnostic Scale, PDS [41]. With respect to the kind of trauma, there was no difference between both patient groups (all *p*>0.1) except for having experienced a life threatening disease, where BPD without PTSD had higher frequencies than those with PTSD (p = 0.009). Also, both patient groups exhibited a similar mean score on the Borderline Symptom List, BSL [42]. BPD and NC women differed on the test of crystallized IQ (WST), which was driven by the BPD with PTSD women. Please refer to Table 1 for a display of patient and control group characteristics.

### 2.2 Ethics Statement

The study was approved by the ethics committee of the Charité Berlin. All participants provided written informed consent.

### 2.3 Neuropsychological Assessment

Crystallized intelligence was assessed by the verbal “Wortschatztest “, WST [43] and fluid intelligence by subtest 4 of the “Leistungsprüfsystem”, LPS [44], a standard German intelligence test. This test shows high validity and a good reliability (retest reliability = .77). On subtest 4, participants have to recognize regularities and irregularities in series of numbers and letters; thus, only minimal education in terms of basic knowledge of numbers and letters is needed. In the standard procedure of the test, as applied in this study, IQ values are adapted for age. The ability to regulate emotions was assessed using the subscale “emotion regulation” of the *Subjective Experience of Emotions* scale (SEE) [45]. The SEE is an established and valid German 42-item-self-report questionnaire (Cronbachs Alpha between.70 und.86, test-retest reliability between.60 and.90), consisting of short one-sentence statements that are rated on a 5 point scale. The emotion regulation subscale consists of 6 sentences (e.g., “If I want to, I can easily manipulate my emotions”, “Most of the time I know how to calm down when I’m heated up”); higher scores indicate better abilities to regulate emotions.

### 2.4 Magnetic Resonance Imaging

Images were acquired on a 1.5-T MRI scanner (Siemens Magnetom Sonata, Erlangen, Germany) with a standard head coil for whole brain MRI data. Two sagittally oriented T1-weighted volumes (TE: 3.56 ms; TR: 12.24 ms; flip angle: 23°; matrix: 256×256; voxel size: 1×1×1 mm) were acquired and used for further processing by the freesurfer image analysis suite (http://surfer.nmr.mgh.harvard.edu/). The freesurfer tool allows quantitative assessment of structural brain data without rater bias.

#### 2.4.1 Cortical thickness measurements

Cortical thickness measurements were carried out as described previously [46]–[48]. After automated processing of the data, the entire cortex of each participant was visually inspected, and inaccuracies in segmentation were manually corrected by persons with extensive training in MRI-based brain anatomical volumetry who were blind to group membership. Freesurfer then generates an output that contains volumetric as well as cortical thickness data for structures predefined by the program (i.e., amygdala, insula). In addition, it provides global thickness data that allow detecting thickness differences in non-predefined regions, searching the entire cortex (i.e., subregions of the DLPFC).

Statistical comparisons of global data and surface maps were generated by computing a general linear model (GLM) of the effects of each variable (group membership, demographic and neuropsychological variables) on thickness at each vertex.

*Non a priori* cortical thickness clusters, which in our case were detected in the DLPFC, were first displayed using qdec (the GUI front end to the statistical engine of freesurfer) with a threshold that shows all vertices with *p-*values between 0.03 and 0.01. To avoid type I error inflation, Monte Carlo simulation was conducted to correct for multiple comparisons on the significant clusters, using a cluster-wise threshold of *p*<0.05. From the corrected clusters, we created an ROI on the group average brain that was mapped back to each individual subject using spherical morphing to find homologous regions across subjects and yield a mean thickness score over the location for each subject.

To validate primary associations between *non a priori* cortical thickness and neuropsychological test scores we took advantage of the built-in function of the qdec freesurfer software, to feed behavioral variables into the GLM. This approach constitutes an unbiased way to look for associations between behavioral variables and cortical thickness across the entire cortex.

With respect to the insula, we used the cortical thickness measure for that structure which was readily generated by the freesurfer parcellation stream in order to establish associations to *non a priori* cortical thickness data of the PFC.

#### 2.4.2 Automated amygdala segmentation

Segmentation of the amygdala was carried out using the freesurfer tool and has been described in detail by Fischl et al. [49], [50]. The resulting volumes were used for the purpose of establishing associations to non a priori cortical thickness data.

### 2.5 Statistical Analysis

Two-tailed independent samples t-tests were used to compare age, IQ, PDS subscores, BSL mean score, and emotion regulation scores between NC and the entire BPD group. To compare NC and BPD subgroups and BPD subgroups to one another on PDS overall traumatic experience, we used univariate ANOVAs with Tukey post-hoc tests. χ^2^ tests were used to compare the groups on discrete variables.

Comparison of DLPFC cortical thickness data was carried out using the GLM within qdec using the Monte Carlo corrected cluster-wide threshold of p<0.05. The thus streamed out data of all BPD patients, BPD without PTSD and NC were compared using two-tailed independent samples t-tests. Cohen’s d was computed to assess effect size of cortical thickness group differences between NC and all BPD patients and NC and BPD without PTSD, with values greater than 0.8 indicating strong effects. Comparison of cortical thickness of the insula and amygdala volume was carried out using univariate ANOVAs with Tukey post-hoc tests. Cohen’s f^2^ was used to assess effect size of insular and amygdalar differences between NC, BPD with PTSD and BPD without PTSD, with f^2^ = 0.02–0,15 indicating small effects. Fisher’s Z was used to compare correlations. All analyses were carried out using the freesurfer tools, respectively and PASW Statistics software package (version 18.0, Chicago, IL, USA).

## Results

### 3.1 Group Differences

#### 3.1.1 Cortical thickness in all BPD patients vs. NC

**Figure 1:**
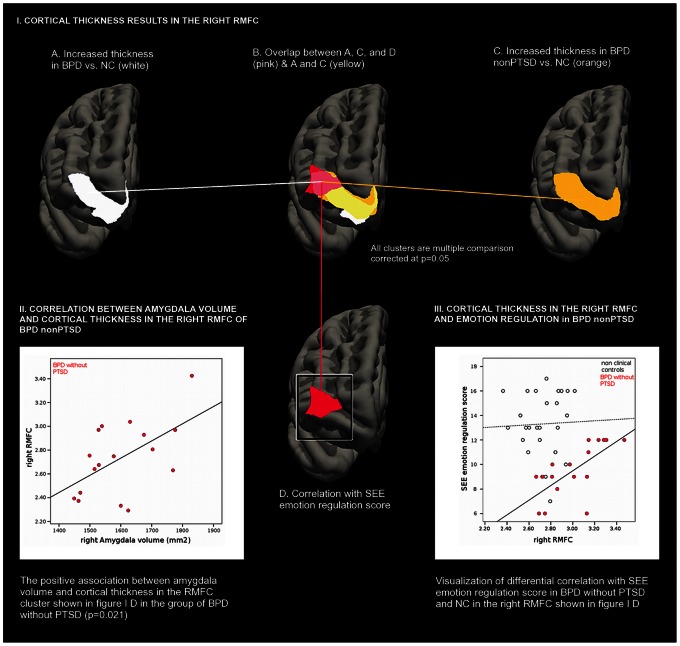
Summary of results.

Cortical thickness was increased in the entire BPD group in a circumscribed cluster (p = 0.05, corrected) located in the right rostral middle frontal cortex, which is part of the DLPFC (RMFC, mean cortical thickness NC: 2.83±0.16 mm vs. BPD: 3.09±0.21 mm) in comparison to NC (d = 1.39). The cluster had a size of 861 mm^2^ and MNI305 coordinates of the maximum were 18.4, 56.3, −14.7 (x,y,z). No significant differences were detected for the left hemisphere. Please refer to figure 1 (panel 1A) for a display of the cluster.

#### 3.1.2 Cortical thickness in BPD patients without PTSD vs. NC

Since our primary goal was to identify brain changes specific to BPD without comorbid PTSD, we then restricted the analysis to those patients. After correction for multiple comparisons, we found increased regional cortical thickness in the right hemisphere, in a virtually identical location to the result of comparing NC to all BPD patients (*p* = 0.05, corrected, *d* = 1.31). Specifically, cortical thickening was detected in a confined cluster of 856 mm^2^ (Figure 1, panel 1C), located within the right RMFC (mean cortical thickness NC: 2.73±0.17 mm vs. BPD without PTSD: 3.01±0.25 mm, MNI305 coordinates of the maximum: *18.8, 56.8, −14.3*). Please refer to figure 1 (panel 1B) for a display of the overlap between the clusters.

#### 3.1.3 Cortical thickness in BPD patients with PTSD vs. NC and in BPD patients without vs. BPD patients with PTSD

No significant differences in cortical thickness were detected for either hemisphere when comparing NC to BPD with PTSD, and when comparing BPD with to BPD without PTSD using qdec within freesurfer.

#### 3.1.4 Cortical thickness of the insula

Neither cortical thickness of the left nor right insula was significantly different between any of the groups (left insula F(2, 55) = 1.353, p = 0.267, f2 = 0.05, and right insula F(2, 55) = 1.701, p = 0.192, f2 = 0.06).

*Right* insula BPD without PTSD: 3.26±0.21 mm, *right* insula BPD with PTSD: 3.16±0.20 mm, *right* insula NC: 3.15±0.20 mm; *left* insula BPD without PTSD: 3.22±0.20 mm, *left* insula BPD with PTSD: 3.20±0.17 mm, *left* insula NC: 3.13±0.20 mm).

#### 3.1.5 Amygdala volume

Neither left nor right amygdala volume was significantly different between the groups (left amygdala F(2, 55) = 1.686, *p* = 0.195, *f^2^* = 0.06, and right amygdala F(2, 55) = 0.553, *p* = 0.578, *f^2^* = 0.02).

*Right* amygdala BPD without PTSD: 1.60±0.12 cc, *right* amygdala BPD with PTSD: 1.62±0.22 cc, *right* amygdala NC: 1.66±0.19 cc; *left* amygdala BPD without PTSD: 1.48±0.13 cc, *left* amygdala BPD with PTSD: 1.42±0.22 cc, *left* amygdala NC: 1.52±0.18 cc).

### 3.2 Brain-Behavior Relationships

To further inform the specific finding of regional cortical thickening in BPD without PTSD (figure 1, panel 1C), in the next step we tested whether there were any associations between the ability to regulate emotions and cortical thickness in BPD without PTSD and NC. Here, we took advantage of the built-in function of the freesurfer software, to feed behavioral variables into the GLM, thus allowing for an independent analysis at the whole brain level. After correcting for multiple comparisons using Monte Carlos simulation with a cluster-wise threshold of *p*<0.05, we found that selectively in a cluster located within the right RMFC (cluster size: 873 mm^2^; mean cortical thickness NC: 2.55±0.15 mm vs. BPD without PTSD: 2.73±0.31 mm; MNI305 coordinates of the maximum: *39.2 48.8 −2.7*), the ability to regulate emotions positively correlated with cortical thickness in BPD without PTSD, but not in NC, showing that cortical thickness in this particular region was associated with emotion regulation abilities specifically in BPD patients without PTSD (NC: r = 0.095, BPD without PTSD^ :^ r = 0.765, Fisher’s z = −2.69, *p = *0.007, see figure 1, panel 1D for the cluster and figure 1 (panel 3) for a visualization of the correlation). Note the overlap between this cluster and the clusters showing the group differences between all BPD patients and NC, and between BPD without PTSD and NC (see figure 1, panel 1B).

To test whether this difference in association can be considered specific to BPD patients without PTSD, we ran an analogous analysis with BPD patients with PTSD and NC. We did not find any significant associations, meaning, no clusters were detected using Monte Carlos simulation with a cluster-wise threshold of *p*<0.05.

We did not detect any significant correlation between amygdala volume and the ability to regulate emotions in any group (NC left: r = 0.007, *p* = 0.973, NC right: r = 0.178, *p* = 0.376, BPD without PTSD left: r = 0.255, *p* = 0.324, BPD without PTSD right: r = 0.380, *p* = 0.132, BPD with PTSD left: r = −0.180, *p* = 0.557, BPD with PTSD right: r = −0.221, *p* = 0.468). Likewise, we did not detect any significant correlation between thickness of the insula and the ability to regulate emotions. (NC left: r = 0.023, *p* = 0.900, NC right: r = 0.237, *p* = 0.235, BPD without PTSD left: r = 0.230, *p* = 0.374, BPD without PTSD right: r = 0.152, *p* = 0.559, BPD with PTSD left: r = 0.099, *p* = 0.747, BPD with PTSD right: r = −0.217, *p* = 0.476).

### 3.3 Brain-Brain Relationships

Because the amygdala has been implicated in emotional dysregulation in BPD [27] and a functional prefrontal-amygdala disconnection has been described in BPD [15], we further explored the relationship between regional cortical thickening in the cluster that had been associated with emotion regulation in BPD without PTSD and amygdala volume in the group of BPD without PTSD [see [46] for a similar approach].

We found that cortical thickness in the RMFC positively correlated with right amygdala volume (r = 0.553, *p = *0.021), see figure 1, panel 2. Since regional cortical thickening had been associated with emotion regulation in independent analyses in the group of BPD without PTSD only, the analysis was restricted to this particular group.

Analogous to the analysis with the amygdala, since the insula has many projections to the PFC and it has been suggested that it might contribute to emotion regulation processes [9], we further scrutinized whether there was any relationship between the thickness of the insula and regional cortical thickening in the cluster that had been associated with emotion regulation in BPD without PTSD. Since that cluster had been associated with emotion regulation in independent analyses in the group of BPD without PTSD only, the analysis was restricted to this particular group. Indeed, we found that the thickness of the left insula positively correlated with cortical thickness in the RMFC (r = 0.857, *p*<0.001).

## Discussion

The primary aim of this study was to identify alterations of brain structure that are specific to BPD without accompanying PTSD. To this end, we used a direct measurement of cortical thickness to be able to catch subtle differences between patients with BPD without PTSD and control groups.

Furthermore, we aimed to scrutinize findings particularly with regard to the current point of view of BPD being associated with a dysfunctional prefrontal-amygdala and prefrontal-insula network underlying the dysregulation of emotion, which is considered to be the core symptom of BPD. Therefore, we sought to ascertain structural correlates of impaired emotion regulation in BPD without PTSD and establish their relationship to the amygdala and insula, respectively.

Our results provide first evidence for increased cortical thickness in the right RMFC, which is part of the DLPFC, in a group of patients with BPD. This finding was specific to those patients without PTSD, as we detected increased cortical thickness only when comparing BPD without PTSD to control subjects, whereas no differences were observed when comparing BPD with PTSD to controls. Furthermore, we show that the increased cortical thickness in the DLPFC of BPD without PTSD is associated with enhanced emotion regulation abilities, commensurate with the assumed role of the DLPFC in emotion regulation. Lastly, since amygdala volume and thickness of the insula in BPD without PTSD were related to cortical thickness in the DLPFC, our findings also provide support for an anatomical basis of an altered fronto-limbic and fronto-insular circuit in the context of emotion regulation in PTSD-free BPD patients.

To the best of our knowledge, this is the first account of cortical thickness in BPD. BPD patients without additional PTSD exhibited increased thickness in a confined area located in the right DLPFC.

Increased rather than decreased cortical thickness in comparison to non-clinical controls has also been reported in other psychiatric populations [46]. In BPD, so far, the DLPFC has been assessed in adults both using manual volumetry (region of interest, *ROI*-based approach) and by using VBM, with no differences to non-clinical controls being reported [21], [22], [24]. The discrepancy between those findings and our results likely stems from both, disentangling the impact of BPD and additional PTSD, as well as employing cortical thickness analysis, which has been shown to be more sensitive to subtle differences than VBM [37] rather than manual or semi-automated techniques.

Moreover, in the ROI-based studies on BPD, differences may have been present but remained undetected due to the size of the ROI. Manual tracing closely follows anatomical landmarks, yielding the volume of a pre-defined structure. Here, we describe alterations manifesting only in part of the DLPFC and not the entire anatomical region, which covers a much bigger volume. Thus, our findings are not discordant with the current literature, but rather add to it by showing that there is an anatomical analog to the aberrant response in the PFC of BPD patients detected by functional imaging studies [29], [51].

Importantly, the alteration in the DLPFC was not observed in patients with an additional diagnosis of PTSD. At first this result seems counterintuitive as one could instead hypothesize that an additional condition would add to the alterations seen in BPD alone. However, several explanatory scenarios are conceivable. First, BPD has some symptomatic overlap with chronic PTSD, e.g., suicidality and self-injurious behavior [52], [53], and due to the diagnostic procedure in DSM IV, mainly relying on behavioral aspects, one could speculate that BPD could be over-diagnosed in the BPD group with PTSD because of this symptom overlap [54]. Hence, the BPD group with PTSD would in fact have a less pronounced form of BPD and would therefore also present with less BPD-specific brain structural alterations. Following this line of reasoning, increased cortical thickness in the group of BPD patients without PTSD could be interpreted as a marker of BPD. A recent study that did not detect cortical thickness differences between patients with abuse-related PTSD and non-clinical controls [55] indirectly supports this interpretation. However, in our dataset, this scenario is rather unlikely, since both BPD groups exhibited a comparable extent of Borderline symptomatology, as evidenced by their BSL scores.

Alternatively, it is conceivable that a premorbid (e.g., genetic) predisposition of some BPD patients could prevent this group from developing comorbid PTSD. Both PTSD and BPD patients are symptomatic survivors of traumatic events, especially childhood sexual abuse [56]–[58]. In our study, both BPD groups experienced equivalent frequency and kinds of trauma. Thus, while being exposed to comparable adversity, one group later develops PTSD and the other one does not. Of note, findings of reduced grey matter density in the DLPFC in teenagers with BPD suggest that the DLPFC is affected early on in the course of the disease [23]. Thus it is conceivable that those patients that do not develop additional PTSD might ultimately present with a more favorable cerebral phenotype, including focally increased cortical thickness, than those that do receive an additional diagnosis, maybe reflecting a premorbid difference.

Another interpretation of our findings could be that increased cortical thickness in the DLPFC of those BPD patients without PTSD might reflect a compensatory mechanism with respect to emotion regulation. In that case, having additional PTSD would interfere with the hypothesized mechanism and the increased cortical thickness seen in patients without PTSD would actually be beneficial. Our finding that increased cortical thickness in the DLPFC was related to better emotion regulation abilities only in BPD patients without PTSD strongly supports this interpretation. The finding that greater cortical thickness in the DLPFC is related to enhanced emotion regulation is also in broad agreement with a structural study, which showed an inverse association between DLPFC volume and impulsiveness in BPD patients [59]. Although we cannot draw conclusions as to the causal relationship between the neuroanatomical finding and affective impairment, the association found here strengthens the argument that BPD is largely conceptualized as a disorder of impaired emotion regulation [1] and that this impairment is reflected on the neuroanatomical level, as well. How exactly increased cortical thickness develops and how this suggested compensation might be operant in BPD would need to be ascertained in future studies, ideally with a longitudinal design.

The DLPFC has been identified to be part of a distributed set of prefrontal regions that together orchestrate the regulation of emotion [60], presumably by regulating the response in limbic areas, such as the amygdala [5], [61]–[63]. This fronto-limbic circuit is assumed to be disturbed in BPD, as an aberrant response of the amygdala [9], [27]–[29] and abnormal PFC functioning [9], [15], [51], [64]–[67] in the context of the processing of emotion and affect have been reported. In addition, it has recently been suggested that the insula might exert modulating effects on emotion regulatory processes involving the PFC [9]. In the present study we also found that amygdala volume and insular thickness were associated with increased cortical thickness in the DLPFC of BPD without PTSD. There are many bidirectional projections between amygdala and PFC [68] and the insula and PFC, respectively [69]. Moreover, emotion regulation has been associated with the relationship between amygdala and PFC [7]. Speculatively, if the increased cortical thickness in the DLPFC indeed reflects a compensatory mechanism for impaired emotion regulation in BPD without PTSD, one would expect that this would also affect the amygdala and insula as part of the regulatory circuit in a beneficial way. Our present results support this assumption, as amygdala volume and insular thickness were positively related to focal cortical thickening, while generally, amygdala volume is reduced in BPD, even in the absence of PTSD [70] and insular volume is decreased in some BPD patients [6], [71].

Taken together, our present results fit well into the framework of impaired PFC-amygdala-insula circuitry in BPD in the context of emotion regulation.

We did not detect cortical thickness differences between BPD patients with and without PTSD. This might have been due to the differences between the two subgroups being more subtle than the differences between the BPD patients without PTSD and controls. Using a larger sample size might reveal those differences.

Interpreting the right-hemispheric lateralization of increased DLPFC thickness is not straightforward, because little prior work speaks directly to this issue in the context of BPD or emotion regulation. Driessen et al. [72] have shown differentially lateralized activation of the PFC in BPD with and without PTSD during the processing of traumatic events. They postulated different neuronal networks within BPD depending on the presence or absence of PTSD, broadly in line with our present findings.

Our study has several strengths. First, in comparison to other studies, we had a relatively large sample size. Second, in our main analyses, we excluded those patients with PTSD, which can be considered a significant confound in other studies on BPD [17]. Lastly, we chose cortical thickness analysis as our main means of assessing the brain, which is geared at detecting even subtle changes in brain anatomy.

However, our study has several limitations. The use of automated volumetric assessment of the amygdala can be considered suboptimal because of the overestimation of volumes in comparison to manual tracing [73]. This might also explain why we did not find the commonly described [19] reduction in amygdala volume. However, our point was not to assess absolute volumes of the amygdala in BPD, but to establish correlations to the PFC, which should be relatively unaffected by this bias. Furthermore, our study design does not permit us to draw conclusions about cause and effect of the relationship between emotional dysregulation and brain alterations. Future studies could address this issue using a longitudinal design with therapeutic interventions aimed at improving emotion regulation capabilities. Lastly, a combination of structural neuroimaging, including DTI, and functional neuroimaging would be desirable to establish a more comprehensive link between the structural alterations we find and emotion regulation in BPD.

In conclusion, we demonstrated increased cortical thickness in a confined area in the right DLPFC in unmedicated women with BPD without comorbid PTSD. This increased cortical thickness was related to enhanced emotion regulation and amygdala volumes, as well as to insular thickness, possibly reflecting a compensatory neural mechanism for emotional dysregulation in BPD.
